# The COVID-19 pandemic and impact on breast cancer diagnoses: what happened in England in the first half of 2020

**DOI:** 10.1038/s41416-020-01182-z

**Published:** 2020-11-30

**Authors:** Toral Gathani, Gill Clayton, Emma MacInnes, Kieran Horgan

**Affiliations:** 1 Cancer Epidemiology Unit, Nuffield Department of Population Health, University of Oxford, Oxford, UK; 2 Department of Breast Surgery, Oxford University Hospitals NHS Foundation Trust, Oxford, Oxford, UK; 3 Department of Breast Surgery, Mid and South Essex NHS Foundation Trust, Chelmsford, UK; 4 Department of Breast Surgery, Bexley Cancer Centre, St James’s University Hospital, Leeds, UK

**Keywords:** Outcomes research, Breast cancer

## Abstract

Delays in cancer diagnosis and treatment due to the COVID-19 pandemic is a widespread source of concern, but the scale of the challenge for different tumour sites is not known. Routinely collected NHS England Cancer Waiting Time data were analysed to compare activity for breast cancer in the first 6 months of 2020 compared to the same time period in 2019. The number of referrals for suspected breast cancer was 28% lower (*N* = 231,765 versus *N* = 322,994), and the number of patients who received their first treatment for a breast cancer diagnosis was 16% lower (*N* = 19,965 versus *N* = 23,881). These data suggest that the number of breast cancers diagnosed during the first half of 2020 is not as low as initially feared, and a substantial proportion of the shortfall can be explained by the suspension of routine screening in March 2020. Further work is needed to examine in detail the impact of measures to manage the COVID-19 pandemic on breast cancer outcomes.

## Background

Concern has been publicly expressed that delays in diagnosis and early treatment for cancer due to the measures instituted to manage the COVID-19 pandemic and the population response to governmental advice to stay at home if possible, will lead to poorer outcomes and shortened survival.^1–3^

In March 2020, a directive was issued to all NHS providers to reconfigure services to manage the anticipated first wave of COVID-19 patients.^4^ Although cancer services were instructed to continue, the imposition of the national lockdown on 23 March 2020, which advised all citizens to stay at home except for exceptional reasons, understandably resulted in a reduction in the numbers of patients accessing healthcare, either through primary care or screening, as patients chose not to present for medical assessment and breast screening units suspended the issuing of routine invitations to reduce footfall in hospitals.

Breast cancer accounts for a third of the cancer incidence in women and a quarter of the total cancer incidence in England, with ~50,000 incident cases diagnosed annually. The overall 5-year survival for the disease exceeds 85%, and less than 15% of disease is locally advanced or metastatic at presentation (stage 3/4).^5^ The significant gains made over the last 20–30 years are largely attributable to early detection and better treatments. The scale of the diagnostic and treatment delay attributed to the pandemic and whether it is equally distributed across all cancer types is currently unknown. Using national data, the numbers of referrals and patients treated for breast cancer in the first half of 2019 and 2020 are reported and compared.

## Methods

Cancer waiting times (CWT) are a measure of National Health Service (NHS) cancer service activity and performance in England. The CWT data are published monthly and provide valuable and contemporary real-world data on referral and treatment.^6^ There are three primary routes to diagnosis for breast cancer; (a) primary care practitioners are encouraged to refer patients with suspected cancer urgently and secondary care should arrange an assessment within 14 days (urgent referrals); (b) In 2010 the Department of Health announced that all patients with breast symptoms should also be seen within 14 days and this lead to a second category of referrals (symptomatic non-urgent) who are also tracked separately on CWT; (c) asymptomatic women aged 50–70 years are diagnosed through the National Health Service Breast Screening Program (NHSBSP).

CWT reports the number of urgent and non-urgent referrals for breast cancer to symptomatic services, and the total number of patients receiving their first definitive treatment for breast cancer, and for how many patients this occurs within 31 days of the decision to treat date, irrespective of the route to diagnosis. This number, reported on a monthly basis, can act as a surrogate for the numbers of newly diagnosed cancers, as a cancer must be diagnosed in order to be treated.

The number of urgent and non-urgent referrals for breast cancer, and the number of patients who received their first treatment for breast cancer in the months of January to June 2019 and 2020 were extracted from the CWT datasets and summarised. The corresponding percentage change of the number of referrals received and the number of patients who received first treatment over the two time periods of interest was calculated.

## Results

The CWT numbers for referral and first treatment activity for breast cancer in England for the first 6 months of 2019 and the first 6 months of 2020 are summarised in Table 1. The total number of referrals to breast cancer diagnostic services decreased by 28% (*N* = 231,765 versus *N* = 322,994) over the first half of 2020 in comparison to the same period in 2019. The observed fall is proportionately much larger in patients referred non-urgently for assessment compared to those referred urgently (40% versus 23%). The reduction was most evident in the months of April and May 2020. The data also show that by June 2020, the numbers of patients referred for urgent assessment was 15% less than compared with June 2019, while the number of non-urgent referrals remained over 40% lower compared to the same month in 2019. The total number of patients receiving their first treatment for breast cancer was 16% (*N* = 3916) lower in the first half of 2020 compared to 2019 (*N* = 19,965 versus *N* = 23,881). The decrease was most evident in the months of May and June 2020. However, the proportion of patients who received their first treatment within 31 days of the decision to treat was on average consistently high (>95%) in the first 6 months of 2020, similar to the corresponding figures for 2019 (>97%).

**Table 1:** National Cancer Waiting Time data for number of referrals and number of patients receiving first treatment for breast cancer in England January to June 2019 and 2020.

Number of referrals	2019	2020	Percentage change in number of referrals 2019/2020		
			All referrals	Urgently referred^a^	Non-urgently referred^a^
January	51,977	50,806	−2.3	+2.0	−11.4
February	52,591	47,868	−9.0	−5.4	−16.9
March	57,953	45,113	−22.2	−19.6	−28.1
April	55,467	20,665	−62.7	−56.6	−77.2
May	53,675	27,784	−48.2	−40.8	−66.0
June	51,331	39,529	−23.0	−14.9	−42.9
Total	322,994	231,765	−28.2	−23.1	−40.2

Number of patients receiving first treatment	2019	2020	Percentage change in numbers of patient receiving first treatment 2019/2020		
January	4227	4194	−0.8		
February	3562	3617	0.0		
March	3861	4990	+ 29.2		
April	3985	3108	−22.0		
May	4208	1930	−54.1		
June	4038	2126	−47.4		
Total	23,881	19,965	−16.4		

^a^The numbers of urgent and non-urgent referrals not shown.

## Discussion

The data presented reflect actual activity in the first 6 months of 2020 compared to the same time period in 2019 rather than a modelled estimate. These data suggest that while there was undoubtedly a marked decrease in the number of referrals made, which may have led to a decrease in numbers of newly diagnosed breast cancers, the magnitude of the decrease in the number of cancers was not as large as initially feared.^7^ The CWT data show that there was a much larger fall in non-urgent referrals for assessment of breast cancer symptoms, where the conversion rate to a cancer diagnosis in 2018/19 was 1.4%, compared to 5.7% for urgent referrals.^8^ Therefore, a reduction in this referral type will translate to a less significant reduction in the overall number of cancers diagnosed.

There is an observed shortfall of just under 4000 breast cancers diagnosed and treated in January–June 2020 compared to 2019. In the 12-month screening cycle of 2018/19, just under 20,000 breast cancers in England were detected through the NHSBSP, which accounted for ~40% of the total breast cancer diagnoses. This equates to ~1600 screen-detected breast cancers per month.^9^ Screening was widely suspended from the end of March 2020, and this suspension will have contributed significantly to the decrease in the number of cancers diagnosed and treated in the second quarter of 2020. The remainder of the observed shortfall will likely be as a combination of cancers that have been diagnosed but not yet treated, and also potentially undiagnosed cancers in the community.

Although surgery is the mainstay of treatment for breast cancer, ~80–85% of breast cancers are responsive to hormonal therapy, which is administered in tablet form and does not require monitoring and does not compromise the immune system. Guidance from professional bodies issued in March 2020 advocated the use of hormonal therapy in patients with hormone positive breast cancer as a temporising alternative to surgery as choice of first treatment to limit hospital exposure for older patients and to assist in managing resource capacity.^10^ This change in practice may explain the spike in the number of women treated in March 2020 in comparison to the same month in 2019 as the prescribing of the hormonal therapy at the diagnostic clinic visit/telephone consultation would have brought forward the date of first treatment rather than waiting for the date of a surgical excision, which would have been the norm in 2019 (Table 1).

Initiatives, such as the B-Map-C audit are attempting to capture and record deviations from standard care in real time and will provide valuable information in due course.^11^

There is a major emphasis in secondary care in the NHS to establish COVID-secure environments, either within the NHS or through the repurposing of private care facilities, to provide safe and timely cancer care that can withstand further waves of the pandemic, and promptly deal with any potential backlogs. Concerns that have been raised about issues such as the potential numbers of undiagnosed screen-detected cancers, and the outcomes of the cohort of breast cancer patients who are receiving endocrine therapy but still require definitive surgical management, need to be addressed. Additionally, the traditional model of breast cancer service delivery via the one-stop clinic, which has a high-volume throughput is challenging to provide in the current era taking into the account of the need for social distancing and the desire to reduce footfall in hospitals. Prior to the pandemic, attendances at such clinics had markedly increased in recent years placing considerable strain on the existing capacity and resources. Research is needed to stratify the cancer risk of patients referred to these services, to allow safe triage of patients in secondary care and to flex capacity in response to demand, and to consider alternate ways of providing safe assessment.

The short- and long-term impact of the decisions made in March 2020 to cope with an unprecedented public health crisis on breast cancer patients will only be fully revealed in the fullness of time.

## Data Availability

All the data used in this study are in the public domain and accessible at https://www.england.nhs.uk/statistics/statistical-work-areas/cancer-waiting-times/.
